# Quantitative Evaluation of Peripheral Arterial Blood Flow Using Peri-Interventional Fluoroscopic Parameters: An In Vivo Study Evaluating Feasibility and Clinical Utility

**DOI:** 10.1155/2020/9526790

**Published:** 2020-01-16

**Authors:** Patrick Ghibes, Gerald Hefferman, Konstantin Nikolaou, Roland Syha, Christoph Artzner, Ulrich Grosse, Rüdiger Hoffmann, Gerd Grözinger

**Affiliations:** ^1^Diagnostic and Interventional Radiology, Eberhard-Karls-University, Tübingen, Germany; ^2^The Warren Alpert Medical School of Brown University, Providence, RI, USA; ^3^Prosper Hospital, Department of Radiology, Recklinghausen, Germany

## Abstract

**Purpose:**

The purpose of this study was to evaluate various objective, quantitative, time-resolved fluoroscopic imaging parameters for use in the peri-interventional evaluation of stenotic peripheral arterial disease lesions. *Material and Methods*. Ten patients (median age, 64; age range, 52 to 79; 8 males, 2 females) with high-grade stenoses of either the superficial femoral or popliteal arteries who underwent endovascular treatment were included. During each intervention, two series of intraprocedural fluoroscopic images were collected, one preintervention and one postintervention. For each imaging series, four regions of interest (ROIs) were defined within the vessel lumen, with two ROIs being proximal (ROIs 1 and 2) and two being distal (ROIs 3 and 4) to the stenosis. The time-density curve (TDC) at each ROI was measured, and the resulting area under the curve (AUC), full width at half maximum (FWHM), and time-to-peak (TTP) were then calculated.

**Results:**

The analysis of the TDC-derived parameters demonstrated significant differences between pre- and postinterventional flow rates in the ROI placed most distal to the stenosis, ROI 4. The AUC at ROI 4 (reported as a relative percentage of the AUC measured at ROI 1 proximal to the lesion) demonstrated a significant increase in the total flow (mean 67.84% vs. 128.68%, *p*=0.003). A significant reduction in FWHM at ROI 4 (mean 2.93 s vs. 1.87 s, *p*=0.003). A significant reduction in FWHM at ROI 4 (mean 2.93 s vs. 1.87 s, *p*=0.003). A significant reduction in FWHM at ROI 4 (mean 2.93 s vs. 1.87 s,

**Conclusion:**

AUC, FWHM, and TTP are objective, reproducible, quantifiable tools for the peri-interventional fluoroscopic evaluation of vessel stenoses. When compared to the standard subjective interpretation of fluoroscopic imagery, AUC, FWHM, and TTP offer interventionalists the advantage of having an objective, complementary method of evaluating the success of a procedure, potentially allowing for more precisely targeted and quantitatively determined treatment goals and improved patient outcomes. This retrospective study was approved by the local ethics committee under the Number 372/2018BO2. The trial was registered at the German clinical trials register under the number DRKS00017813.

## 1. Introduction

Peripheral arterial disease (PAD) is widespread in developed countries and is increasing in both incidence and prevalence worldwide [1–4]. The characteristic vascular lesions of PAD lead to vessel lumen narrowing and stenosis, resulting in diminished blood flow distal to the lesions and clinical complaints ranging from mild claudication to critical limb ischemia. A range of imaging modalities are employed in the evaluation of these stenotic lesions, including color-coded duplex sonography (CCDS), contrast-enhanced computed tomography angiography (CTA), magnetic resonance angiography (MRA), and digital subtraction angiography (DSA).

DSA is presently considered the gold standard for both PAD diagnosis and stenosis grading; it is associated with the highest sensitivity and specificity for the detection of stenotic lesions and provides detailed morphologic and dynamic flow information. Additionally, during catheter-directed DSA imaging, endovascular treatments including balloon angioplasty and stent placement can be simultaneously performed [1, 5]. Despite these important advantages, the imaging data provided by the conventional DSA do not directly allow for quantitative measurement of flow conditions or changes in flow, forcing interventionalists to rely exclusively on their subjective impressions of contrast flow during image-guided interventions [3, 5]. As a consequence, intraprocedural determination of treatment success is defined by subjective assessment rather than the attainment of a quantitative blood flow target. While both MRA and CCDS can provide quantitative flow measurements, neither of them can be efficiently implemented intraprocedurally, making them an impractical method of defining treatment success for the interventionalist [6].

The recent development of quantitative, color-coded fluoroscopy and DSA provides novel techniques with the potential to surmount this important clinical limitation. Both techniques allow for the intraprocedural generation of time-density curves (TDCs), defined as the measured average radiodensity at predefined regions of interest (ROIs) as a function of time as a bolus of iodinated contrast passes within the target vessel lumen. From these TDCs, a diverse set of flow parameters can be derived, specifically area under the curve (AUC), full width at half maximum (FWHM), and time-to-peak (TTP), each with potential clinical utility as a means of characterizing intraluminal blood flow.

Several studies have demonstrated that color-coded quantitative DSA is a useful tool in the evaluation and treatment of neurovascular pathology; however, the application of the technique to PAD stenoses remains experimental and is not a part of clinical routine [7–11]. In cases of PAD lesions affecting the superficial femoral artery (SFA) and popliteal artery (PA), investigations have been limited to comparing quantitative, color-coded DSA against CCDS and nonimaging modalities, specifically ankle-brachial index (ABI) [6, 12]. Importantly, these studies limited their investigation to the evaluation color-coded DSA, which uses frame rates and order of magnitude slower (3-4 frames/s vs. ≥30 frames/s) and radiation exposure and order of magnitude higher (∼1 mSv/min vs. ∼0.1 mSv/min) than quantitative fluoroscopy, resulting in coarse time resolution and increased exposure to ionizing radiation [13]. In contrast, TDC-derived flow parameters measured via quantitative fluoroscopy have received no direct clinical investigation in the context of peripheral arterial lesions; thus, there exists a substantial possibility that the full clinical utility of quantitative fluoroscopy has yet to be utilized.

The aim of this in vivo study was to directly investigate the feasibility and reproducibility of these fluoroscopically measured TDC-derived flow parameters as a means of quantitatively evaluating treatment success in the context of PAD lesions affecting the SFA and PA. Specifically, AUC, FWHM, and TTP were investigated as a means of quantitatively evaluating changes in blood flow across vessel stenoses during endovascular treatment.

## 2. Material and Methods

A total of 10 patients (median age, 64; age range, 52 to 79; 8 men, 2 women) with PAD who underwent fluoroscopically guided endovascular intervention between November 2016 and February 2018 were included in this retrospective, institutional review committee-approved study. Patients with symptomatic stenosis (Fontaine category 2b to 4) of the superficial femoral artery (*n* = 6) or popliteal artery (*n* = 4) and with no contraindications to revascularization procedures (e.g., impaired renal function, unstable vital signs) were included. Hemodynamic stenosis grading was confirmed by Doppler ultrasound examination and ankle-brachial index measurement. Detailed patient information is further summarized in Table 1.

All endovascular interventions were performed using a single flat-panel robotic angiography system (Artis Zeego Q, Siemens Healthcare GmbH, Erlangen, Germany). After positioning the patient in a supine position on the angiography suite table, the arterial system was accessed via puncture of the common femoral artery. A 6-F sheath (Radiofocus/Destination, Terumo, Leuven, Belgium) was then introduced in either the antegrade direction toward the lesion or using a crossover maneuver. Fluoroscopic imaging was conducted using a single injection of 10 mL of diluted contrast agent (5 mL saline solution and 5 mL Ultravist 370 (Bayer HealthCare LLC, Whippany, NJ, USA) or Imeron 400 (Bracco UK Ltd., Buckinghamshire, UK). The diluted contrast agent was applied manually within two seconds. The start of image acquisition was manually triggered, and the acquisition was stopped after the signal loss of the contrast agent bolus. The frame rate of each fluoroscopic imaging series was 30 images per second. Two interventional treatment options were employed at the discretion of the operator: angioplasty using drug-eluting balloons or stent placement. At the conclusion of the initial intervention, a postinterventional image series was acquired before procedure completion. Patients with residual stenoses despite drug-eluting balloon angioplasty (*n* = 4) underwent stent placement and an additional fluoroscopy image series was obtained. The pre- and posttreatment fluoroscopic image series were then saved and exported for analysis.

**Figure 1:**
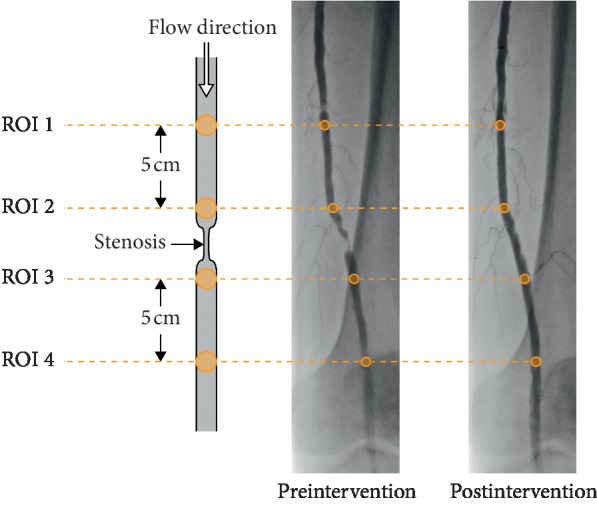
Schematic illustration of the positions of ROIs 1–4 relative to each other and to the stenosis.

For postprocessing and data analysis, ImageJ (US National Institutes of Health, Bethesda, Maryland, USA), a computer program that enables both the color-coded visualization of contrast agent flow and the calculation of the TDCs at preselected ROIs, was used. TDCs calculations were performed using four ROIs for each image series, with two positioned proximally (ROIs 1 and 2) and two distally (ROIs 3 and 4) relative to the lesion. This ROI positioning scheme is illustrated in Figure 1. Each ROI was manually placed in order to fill the vessel diameter completely. The position of a ROI was adapted in cases where vessel anomalies or vessel branch points were at the intended position. The first step of the postprocessing workflow was to place ROIs 1 through 4 in a patient image series and to calculate the resulting TDCs. The ROI positions in the preinterventional image series were then transferred to the postinterventional image series in order to maximize comparability between images. Signal changes caused by X-ray absorption from the patient rather than the contrast bolus were eliminated by setting the initial value before contrast arrival to zero.

**Figure 2:**
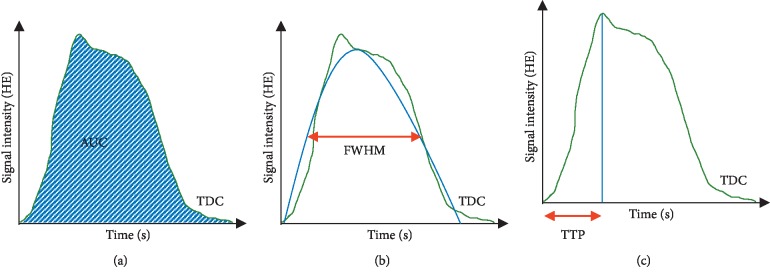
Graphics illustrating the measurement parameters derived from TDCs: (a) area under the curve (AUC); (b) full width at half maximum (FWHM); (c) time-to-peak (TTP).

The measured TDCs were then used to calculate the three derived measurement parameters used in this study—AUC, FWHM, and TTP—which are illustrated in Figure 2. The measurement parameter AUC was defined as the integral of the time-dependent mean radiodensity at an ROI, caused by the contrast agent bolus passage. Calculation of AUC was performed using QTI Plot computer software (QTI Plot, IONDEV SRL, Bucuresti, Romania). The summation integral began when the average signal in the ROI increased due to the contrast agent bolus administration, and the total integration time was kept constant for pre- and postinterventional image series of equal length. The resulting AUCs in ROIs 2–4 were then reported as a percentage of the AUC observed in ROI 1; these percentage values were then used to compare the pre- and postinterventional AUC results at each ROI. FWHM was calculated by fitting Gaussian curves to the TDC data using QTI Plot software. FWHM was defined as the contiguous length of time at which the value of the resulting Gaussian curve was at least half of its maximum value. TTP was defined as the length of time between contrast bolus arrival at a ROI (i.e., the start of the initial upslope in the corresponding TDC) and the TDC reaching its maximum value. Some TDCs demonstrated plateau phases with multiple peaks due to flow changes induced by the cardiac cycle; in such cases, the first peak was chosen.

All statistics were performed using the statistical software SPSS (IBM Corporation, Armonk, NY, USA) and Stata (College Station, Texas, USA). For all TDC-derived parameters (AUC, FWHM, and TTP), the arithmetic mean and standard deviation for descriptive statistics were calculated. Pre- and postinterventional differences in these parameters were calculated at each ROI; statistical comparisons were made only between the pre- and postinterventional values at each ROI, not between differing ROIs. Significant differences between mean values were evaluated via the nonparametric Wilcoxon signed-rank test. Correlations of the deviations in the TDC-derived parameters at each ROI across all patients before and after intervention were calculated using Spearman rank correlation coefficients (rho). A *p* value <0.05 was considered significant.

## 3. Results

Overall, ten patients with stenosis of the superficial femoral artery or popliteal artery were treated. All patients had a single short (<6 cm length) high-grade (>75% luminal obstruction) stenosis. All patients had a minimum of one patent run-off vessel to the foot that was free of clinically relevant stenosis. Nine patients underwent angioplasty of the index lesion using drug-eluting balloons, and 6 patients received stent implantation after angioplasty. Revascularization was successful for all patients without any subjective visual signs of residual stenosis evident in control DSA images. One minor complication occurred (a pseudoaneurysm at the puncture site), and no major complications were encountered.

### 3.1. AUC

**Figure 3:**
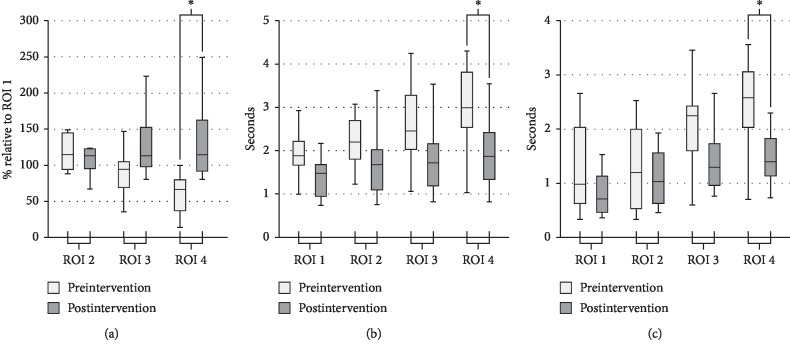
Box plots displaying pre- and postinterventional results at ROIs 1–4 derived from TDC data: (a) area under the curve (AUC); (b) full width at half maximum (FWHM); (c) time-to-peak (TTP).

Table 2 and Figure 3(a) illustrate pre- and postinterventional AUC results. Percentages in ROIs 2–4 relative to reference ROI 1 are reported. A significant increase from pre-to postinterventional AUC was observed at ROI 4 (67.84% vs. 128.68%, *p*=0.003), while no significant changes were observed at ROI 2 (117.01% vs. 112.74%, *p*=0.579) or ROI 3 (92.59% vs. 124.69%, *p*=0.105). Correlation between percentage AUC deviation from pre-to postintervention across all 10 patients revealed a strong correlation for ROI 4 (rho = 0.659, *p*=0.002) and no significant correlations for ROI 2 (rho = −0.139, *p*=0.560) or ROI 3 (rho = 0.382, *p*=0.097).

### 3.2. FWHM

Table 3 and Figure 3(b) illustrate the pre- and postinterventional results for FWHM. As a general trend, FWHM was observed to have decreased in all ROIs after the endovascular intervention. A significant difference was observed at ROI 4 (2.93 s vs. 1.87 s, *p*=0.015), while no significant changes were observed at ROI 1 (1.95 s vs. 1.53 s, *p*=0.089), ROI 2 (2.19 s vs. 1.69 s, *p*=0.105), or ROI 3 (2.58 s vs. 1.78 s, *p*=0.070). A preinterventional outlier value was noted in one patient at ROI 3; consequently, pre- and postinterventional FWHM data from ROI 3 of this patient were excluded from the analysis. A strong correlation was demonstrated at ROI 4 (rho = −0.555, *p*=0.011), a moderate correlation was observed at ROI 3 (rho = −0.486, *p*=0.030), and no significant correlations were observed at ROI 2 (rho = −0382., *p*=0.097) or ROI 1 (rho = 0.399, *p*=0.081).

### 3.3. TTP

Table 4 and Figure 3(c) illustrate the pre- and postinterventional results for TTP. Similar to the results observed for FWHM, all postinterventional TTP results demonstrated a decreasing trend from their respective preinterventional values. A significant decrease was observed at ROI 4 (2.43 s vs. 1.45 s, *p*=0.009), while no significant changes were observed at ROI 1 (1.26 s vs. 0.82 s, *p*=0.280), ROI 2 (2.07 s vs. 1.40 s, *p*=0.063), or ROI 3 (2.07 s vs. 1.40 s, *p*=0.063). A strong correlation was observed at ROI 4 (rho = −0.590, *p*=0.006), and no significant correlations were observed at ROI 1 (rho = 0.260, *p*=0.268), ROI 2 (rho = −0.121, *p*=0.610), or ROI 3 (rho = −0.434, *p*=0.056).

Table 5 summarizes all values for AUC, FWHM, and TTP at ROI 4 for each patient. Values demonstrating postinterventional improvement are marked. For each patient, at least one parameter was observed to have improved; 7 of 10 patients (70%) showed improvement in all three parameters, 2 patients (20%) showed improvement in 2 of 3 parameters, and 1 patient (10%) showed improvement in 1 of 3 parameters.

## 4. Discussion

In clinical practice, nearly all endovascular procedures performed for the treatment of PAD are guided using a combination of DSA and fluoroscopic imaging. During these procedures, stenosis grading and assessment of blood flow are based on the rough visual estimation of contrast agent bolus flow by the interventionalist using two-dimensional image series. This technique remains the current gold standard, despite the fact that the accuracy of the visual estimation of peripheral vessel stenoses is known to be limited and the degree of stenosis is often overestimated [14]. These problems are further compounded by the fact that the degree of stenosis may be misinterpreted based on the angle of projection of the two-dimensional DSA images. Invasive measurement of the pressure gradient across a vascular lesion is a potential solution to this problem; however, this procedure is time-consuming and necessitates the use of additional equipment, substantially reducing its clinical utility as a standard means of assessing treatment success. As a consequence, there is a clinical need for novel, quantitative functional parameters to aid the interventionalist in quickly and objectively evaluating the hemodynamic significance of vascular lesions.

In recent years, software solutions that can be used to calculate intraluminal blood flow parameters based on DSA and fluoroscopic image series in order to quantitatively evaluate treatment success have been developed. To date, color-coded quantitative DSA, rather than quantitative fluoroscopy, has been the subject of all clinical investigations resulting from these technical advances. These studies have investigated a variety of functional parameters, with the aim of extracting objective hemodynamic data from DSA imaging during peripheral arterial and cerebrovascular interventions [6, 9, 12, 15–19]. Kostrzewa et al. investigated FWHM and TTP as parameters for treatment evaluation of SFA and AP lesions, demonstrating that both parameters could be used to assess treatment success independent of ABI value [6]. Lou et al. observed similar results using TTP in the evaluation of hemodynamic changes and treatment success for lower extremity arteries [15]. A related alternative approach was investigated by Kim et al., who demonstrated that the color-coded DSA-perfusion parameters AUC, TTP, and arrival time of contrast agent in the distal lower extremity are sensitive methods of treatment assessment in patients with upstream vessel stenoses [12]. These and similar studies provide increasing evidence that the quantitative evaluation of hemodynamic change is a viable means of peri-interventionally assessing treatment success in patients with arterial lesions.

This study adds to this body of evidence and expands it to include quantitative blood flow parameters derived from fluoroscopic imaging, rather than DSA alone. Quantitative fluoroscopy offers several key benefits over DSA, including increased time resolution and reduced exposure to ionizing radiation. Increased time resolution, in particular, has the potential to offer a uniquely valuable improvement over alternative quantitative image-based methods, a consequence of fluoroscopy's order-of-magnitude increase in frame rate relative to DSA. This potential improvement is due to the fact that all quantitative image-based methods are fundamentally reliant on calculations derived from TDCs; by providing superior time resolution, clinically relevant variations in TDC data are better characterized, a benefit that extends to all TDC-derived parameters. It is important to note, however, that the goal of this preliminary study was to establish the clinical utility of these fluoroscopically generated TDC-derived parameters, rather than a direct comparison of TDC-based parameters gathered via quantitative DSA versus quantitative fluoroscopy. Based on the results of this study, which affirm this potential clinical utility, future work directly comparing the sensitivity, specificity, and related clinical parameters of DSA-based versus fluoroscopy-based TDC-derived parameters is warranted and will be the subject of future investigation.

**Figure 4:**
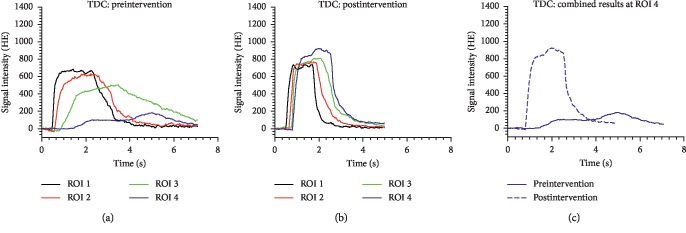
Time-density curves (TDCs) for ROIs 1–4 of an exemplary patient (63 years old, Fontaine 2B stenosis of superficial femoral artery): (a) flow conditions prior to intervention; (b) flow conditions after balloon angioplasty and stent placement; (c) pre- and postinterventional results at ROI 4.

The results of this study show that fluoroscopically measured AUC, FWHM, and TTP all demonstrate significant pre- to postinterventional changes at ROI 4 distal to the stenosis. A significant increase in AUC was observed at ROI 4, a result that is consistent with recent related work [12]. AUC represents the sum of the signals in a ROI, which generated the contrast agent flow. Low flow over the stenosis, jet phenomena, turbulent flow, and shunting to collateral pathways all result in a preinterventional reduction in AUC at the ROIs distal to the stenosis, especially ROI 4 [20–22]. Postinterventional fast flooding of contrast agent bolus and redistribution of blood away from collaterals and toward the major arterial flow-path lead to a significant increase of the signal in ROI 4, as demonstrated in Figure 4(c), an effect predicted in prior studies using phantom arterial models [22].

Similarly, the significant pre- to postinterventional change in FWHM at ROI 4 corresponds to a newly unobstructed flow of the contrast agent bolus throughout the treated stenosis. As compared to nonstenotic areas, a contrast bolus traveling through a stenosis requires more time to move distally, broadening the TDC and increasing FWHM. These results are similar to those observed in previous studies using DSA rather than quantitative fluoroscopy [6, 12]. These data also complement the work conducted by Kim et al., who demonstrated that DSA-based FWHM was a viable method of quantifying distal tissue profusion of the lower leg as a means of establishing successful intervention at the feeding artery, providing further evidence of the validity of TDC-derived parameters as a means of evaluating PAD treatment success [12].

The results regarding TTP similarly demonstrate its suitability as a surrogate marker for flow improvement. It is important to note that, in some patient cases, differences between preinterventional TTP and postinterventional TTP are small; therefore, TDC peaks must be selected with care. In such cases, the novel quantitative fluoroscopy-based approach employed in this study offers a substantial advantage over prior investigations due to the much-improved temporal resolution as compared to DSA data.

The results of this study also demonstrate that, in 30% of patients, at least one parameter failed to show improvement after successful intervention. Consequently, it will likely be necessary to simultaneously utilize more than one parameter for quantification of treatment success in clinical practice. The fact that at least one parameter changed in all of the patients makes these parameters promising tools for a broader in vivo application; however, a further prospective investigation is necessary in order to better define the sensitivity and specificity of these parameters and to determine the optimal set of parameters to utilize in clinical practice.

Overall, these results demonstrate that TDC-derived quantitative flow parameters have considerable potential as tools to determine the success of endovascular interventions. This potential is perhaps best illustrated by quantitatively determining whether balloon angioplasty alone is sufficient for the treatment of a stenotic lesion, or if further intervention with stenting is warranted. Patients could thus be prevented from receiving potentially unnecessary stenting and the resulting antithrombotic therapy it necessitates, while patients requiring additional treatment can be intervened on immediately and spared the need for later reintervention.

The quantitative parameters investigated in this study additionally have expanded potential as a means of characterizing the iatrogenic dissections that can complicate endovascular interventions. These dissections often have deleterious hemodynamic effects and can negatively impact patient outcomes; as a consequence, characterizing whether an angiographically apparent dissection is flow-limiting and thus requires additional intervention is an important area of potential patient benefit [23]. Similar to the inaccuracies which result from a purely subjective assessment of stenoses, subjective judgement of dissection severity based on visual DSA imaging alone is challenging, and dissection severity is often underestimated [24]. Thus, the quantitative parameters described in this investigation may additionally provide important clinical information regarding the degree of dissection and the need for additional intervention. This line of investigation was beyond the subject of this study, however, and will be investigated further in future work.

This study has several limitations. The main limitation is the small sample size (*n* = 10). Despite this small sample, the statistical evaluation of the results demonstrated significant improvements in ROI 4 for all three parameters. A second limitation is that all ROIs were placed by hand; consequently, ROI size and position were the result of the specific choices of the operator. Future software solutions that combine automatic vessel diameter determination and motion correction may play a major role in future measurement standardization; at present, however, all vessel segmentation algorithms still require operator placement and confirmation, particularly as the calcified vessel walls common in patients with PAD can lead to the mischaracterization of vessel diameter.

## 5. Conclusion

The results of this investigation demonstrate that the fluoroscopically measured, TDC-derived flow parameters AUC, FWHM, and TTP have substantial potential as a means of quantifying the effective restoration of flow across PAD stenoses. Measurement of these parameters requires only software-based processing of existing angiographic data and does not require any additional invasive measurement, ionizing radiation exposure, or contrast administration beyond that of established endovascular treatment techniques. Consequently, further investigation into the use of these objective measures—particularly the establishment of quantitative, clinically significant flow improvement goals—should be actively undertaken.

## Figures and Tables

**Table 1:** Study patient and lesion characteristics: BMI, body mass index; SFA, superficial femoral artery; PA, popliteal artery.

Data		Value
Age	Mean ± standard deviation	64.21 ± 11.67

Sex	Male	8
	Female	2

BMI	Mean ± standard deviation	29.32 ± 2.16

Fontaine classification	2b	8
	4	2

Stenosis location	SFA	6
	PA	4

TASC classification	A	5
	B	4
	C	1

**Table 2:** AUC data before and after intervention for ROIs 2 to 4 as compared to reference ROI 1 (defined as having an AUC of 100%).

ROI	AUC: preintervention (%)	AUC: postintervention (%)	Significance of change (*p* value)	Correlation coefficient (rho)	Significance of correlation (*p* value)
I	100	100	—	—	—
II	117.01 (±25.67)	112.74 (±33.80)	0.579	−0.139	0.560
III	92.59 (±55.0)	124.69 (±43.10)	0.105	0.382	0.097
IV	67.84 (±45.72)	128.68 (±53.13)	0.003^*∗*^	0.659	0.002^*∗*^

The arithmetic mean plus/minus standard deviation and *p* values demonstrating the significance of pre- to postinterventional changes are listed. Spearman rank correlation coefficients (rho) and corresponding *p* values demonstrating the significance of correlations in changes across all 10 patients at each ROI are also listed. *p* values are calculated based on changes at each specific ROI and do not compare differing ROIs to one another. Significant values are marked with an asterisk.

**Table 3:** FWHM data before and after intervention for ROIs 1 to 4.

ROI	FWHM: preintervention (s)	FWHM: postintervention (s)	Significance of change (*p* value)	Correlation coefficient (rho)	Significance of correlation (*p* value)
I	1.95 (±0.66)	1.53 (±0.75)	0.089	−0.399	0.081
II	2.19 (±0.64)	1.69 (±0.78)	0.105	−0.382	0.097
III	2.58 (±0.97)	1.78 (±0.84)	0.070	−0.486	0.030^*∗*^
IV	2.93 (±1.05)	1.87 (±0.81)	0.015^*∗*^	−0.555	0.011^*∗*^

The arithmetic mean plus/minus standard deviation and *p* values demonstrating the significance of pre- to postinterventional changes are listed. Spearman rank correlation coefficients (rho) and corresponding *p* values demonstrating the significance of correlations in changes across all 10 patients at each ROI are also listed. *p* values are calculated based on changes at each specific ROI and do not compare differing ROIs to one another. Significant values are marked with an asterisk.

**Table 4:** TTP data before and after intervention for ROIs 1 to 4.

ROI	TTP: preintervention (s)	TTP: postintervention (s)	Significance of change (*p* value)	Correlation coefficient (rho)	Significance of correlation (*p* value)
I	1.26 (±0.75)	0.82 (±0.41)	0.280	−0.260	0.268
II	1.32 (±0.77)	1.11 (±0.53)	0.631	−0.121	0.610
III	2.07 (±0.78)	1.40 (±0.58)	0.063	−0.434	0.056
IV	2.43 (±0.88)	1.45 (±0.48)	0.009^*∗*^	−0.590	0.006^*∗*^

The arithmetic mean plus/minus standard deviation and *p* values demonstrating the significance of pre- to postinterventional changes are listed. Spearman rank correlation coefficients (rho) and corresponding *p* values demonstrating the significance of correlations in changes across all 10 patients at each ROI are also listed. *p* values are calculated based on changes at each specific ROI and do not compare differing ROIs to one another. Significant values are marked with an asterisk.

**Table 5:** Changes in AUC, FWHM, and TTP at ROI 4 for each of the 10 patients included in this study.

Patient number	AUC (% vs. ROI 1)		FWHM (s)		TTP (s)	
	Pre	Post	Pre	Post	Pre	Post
1	172.89	117.96^*∗∗*^	1.03	0.82^*∗*^	0.70	0.95^*∗∗*^
2	75.36	162.41^*∗*^	3.65	1.97^*∗*^	2.70	1.67^*∗*^
3	24.35	249.70^*∗*^	2.63	1.97^*∗*^	2.40	1.20^*∗*^
4	80.00	94.091^*∗*^	2.54	3.55^*∗∗*^	2.03	1.83^*∗*^
5	13.55	173.52^*∗*^	3.85	1.34^*∗*^	3.56	1.23^*∗*^
6	43.00	111.11^*∗*^	2.63	1.85^*∗*^	2.46	1.13^*∗*^
7	73.69	80.69^*∗*^	3.36	2.42^*∗*^	3.13	1.93^*∗*^
8	99.88	80.62^*∗∗*^	3.81	2.49^*∗*^	3.06	2.30^*∗*^
9	59.22	125.11^*∗*^	1.56	0.90^*∗*^	1.30	0.73^*∗*^
10	36.48	91.41^*∗*^	4.30	1.90^*∗*^	3.03	1.56^*∗*^

Values for pre- and postinterventional measurements are listed. Postinterventional values that demonstrate improvement are denoted by asterisk; values that do not demonstrate improvement are denoted with double asterisk.

## Data Availability

The data used to support the findings of this study are available from the corresponding author upon request.
